# Influenza-associated hemolytic uremic syndrome: The pathogenic role of the virus 

**DOI:** 10.5414/CNCS110219

**Published:** 2021-04-16

**Authors:** Valeria Silecchia, Gianluca D’Onofrio, Enrico Valerio, Giulia Rubin, Enrico Vidal, Luisa Murer

**Affiliations:** 1 Department of Pediatrics, Ospedale “Dell’Angelo” Mestre Venice,; 2 Pediatric Nephrology, Dialysis, and Transplantation Department of Woman and Child Health, University of Padova, Padova,; 3 Department of Pediatrics, Ospedale “San Bortolo” Vicenza, and; 4 Department of Pediatrics, Ospedale “Santa Maria della Misericordia” University of Udine, Udine, Italy

**Keywords:** hemolytic uremic syndrome, H1N1, pathogenesis, complement

## Abstract

A 3-year-old girl came to our attention for fever and upper respiratory tract infection associated with thrombocytopenia, non-immune hemolytic anemia, and acute kidney injury (AKI). Complete blood count and renal function slowly normalized, with no need for dialysis. She was always normotensive with valid diuresis; her neurological status also rapidly improved. Nasal swab turned out positive for influenza A H1N1; stool test was negative for Shiga toxin-producing *Escherichia coli* (STEC). The patient was treated with oseltamivir for 5 days with a favorable outcome. Association between hemolytic uremic syndrome (HUS) and H1N1 influenza is poorly reported in literature [1, 2, 3, 4]. The pathogenic role of the virus in causing HUS is still controversial and debated [1, 2, 3, 4]. In our patient, complement activity markers (serum C3 and C5b-9) alteration suggested a transient, virus-mediated complement activation.

## Introduction 

Hemolytic uremic syndrome (HUS) is characterized by the clinical triad of hemolytic anemia, thrombocytopenia, and acute renal failure [5]. Two forms of HUS are classically known: typical, caused by STEC and atypical (aHUS), caused by mutation of complement regulatory genes, anti-factor B and H complement antibodies, DGKE mutations, congenital defects of cobalamin metabolism or secondary to pregnancy, drug use, or infections (*Clostridium perfringens*, *Streptococcus pneumoniae*, HIV, coxsackievirus, EBV, varicella, and influenza virus) [6]. The association between aHUS and influenza A H1N1 infection is documented in literature, even though not many cases are reported [1, 2, 3, 4]. It is still under debate whether the virus could merely represent a trigger or it could have a direct mechanism in the pathogenesis of aHUS [1, 2, 3, 4]. 

## Case report 

We evaluated a 3-year-old girl for 48 hour-lasting high fever associated to rhinitis and catarrhal cough. Personal history was unremarkable, except for a neonatal sepsis. She did not complain about gastrointestinal symptoms; there was no history of ingestion of potentially contaminated food. Physical examination showed widespread petechiae over limbs, face, and back. The patient was irritable and drowsy at presentation. She was normotensive, moderately dehydrated with conserved urinary output. Lab tests showed thrombocytopenia (16,000/mm^3^), hemolytic anemia (Hb 10.5 g/dL, haptoglobin 0.25 g/L, lactate dehydrogenase 2,232 U/L with negative indirect Coombs test) with 3% schistocytes on the peripheral smear; acute renal failure (urea 104 mg/dL, creatinine 1.2 mg/dL); mild rise of inflammatory markers (CRP 11.3 mg/L, PCT 9.91 mg/mL); hypoalbuminemia (2.3 g/dL); elevated D-Dimer concentration (18733 ng/mL), no fibrinogen consumption (162 mg/dL), and normal clotting (INR 1.27, aPTT ratio 1.35). Urinalysis detected proteinuria (1 g/dL) and hemoglobinuria (0.5 mg/dL). Admission chest X-ray was normal; abdomen ultrasound scan showed bilateral hyperechogenicity of renal cortex, with reduced corticomedullary differentiation. Stool cultures excluded a STEC infection. A working diagnosis of aHUS was then made. Molecular tests were carried out to determine dysregulations of the alternative pathway of complement (APC): C3 was moderately low (0.52 g/L), C4 was normal and C5b-9 was mildly increased either on resting (190%, reference range (r.r.) < 150%) and ADP-activated endothelial cells (205%, r.r. < 150%). Autoimmunity markers (ANA, antiDNA antibodies) were negative. Microbiologic work-up revealed influenza A H1N1 positivity by PCR on nasal swabs, thus suggesting the diagnosis of influenza A-related aHUS. Given the patient’s neurologic symptoms at presentation (drowsiness, irritability), we performed differential with thrombotic thrombocytopenic purpura (TTP): ADAMTS-13 activity was normal (77%, r.r. 65 – 130) and anti-ADAMTS-13 antibodies were negative. Initial infusion of half-normal saline (NaCl 0.45%) was gradually tapered, and albumin was started. Diuresis, monitored by bladder catheter, always remained valid with good blood pressure control. Serum creatinine peaked on the third day (1.9 mg/dL, estimated GFR 9 mL/min/1.73m^2^ with bedside Schwartz formula (BSF)) with subsequent spontaneous drop, with no need for dialysis. Given influenza A positivity, oseltamivir was started and maintained for 5 days, with dose adjustment according to renal clearance. Fever ceased after 72 hours of antiviral therapy. Initial drowsiness and irritability rapidly disappeared, with persistently negative neurological examination afterwards. Two packed red blood cell transfusions and a platelet transfusion for clinical epistaxis were needed. At discharge, eGFR (BSF) was 35 mL/min/1.73m^2^ with nearly physiological values of proteinuria (14 mg/kg/day). At 7-day follow-up, the patient had fully recovered with complete normalization of hematologic and renal parameters. 

## Discussion 

Historically, many infectious agents have been linked to aHUS. While the association of pneumococcal infection and HUS is well documented in the literature [7], only a few cases of aHUS have been described in association with influenza A virus H1N1 [1, 2, 3, 4]. These cases have been reported both in association with mutations of complement regulation factors, suggesting a trigger role for the virus, and in absence of genetic predisposing factors [8]. A potential direct pathogenicity of the H1N1 virus has been hypothesized on the basis of in vitro and in vivo studies showing viral capability to determine endothelial cell apoptosis, platelet activation and therefore the formation of microthrombi [9, 10, 11]. The pathogenicity of the influenza virus also depends on the ability to produce neuroaminidase (NA), similarly to *Streptococcus pneumoniae*. The pneumococcal circulating NA cleaves the sialic acid residues of membrane glycoproteins of red blood cells, platelets and glomerular endothelial cells, determining the exposure of the T antigen that binds IgM with consequent antibody-mediated injury [12]. However, influenza virus produces less NA than pneumococci, and viral NA is membrane-associated rather than circulating, so its pathogenetic contribution to HUS seems different. Actually, influenza virus-mediated desialylation of cell membrane glycans is associated with vigorous deposition of C3b and consequent alternative pathway activation [13]. Furthermore, Berdal et al. [14] reported an increase in plasma C5b-9 levels in patients with influenza A H1N1 and Sun et al.’s [15] study showed that lung injury in influenza A-infected mice was linked to excessive complement activation with deposition of C3 and C5b-9 and an increased expression of complementary receptors C3aR and C5aR. Therefore, influenza virus could act similarly to VTEC, stimulating the expression of adhesion molecules capable of transient activation of the APC. In fact, in VTEC infection the role of P-selectin, one of the up-regulating adhesion molecules from shigatoxin, which acts as a C3b receptor, stabilizes C3-convertase, and triggers the APC, is well known [16]. The existence of an analogous adhesion molecule linking the action of the Influenza virus to the activation of APC remains hypothetical. Previous studies hypothesize a viral trigger action on a HUS-susceptible haplotype [17, 18, 19]. In Bitzan’s survey, some patients with thrombotic microangiopathy associated with influenza presented genetic mutations of complement (C3 and MCP mutations) [6]. This association could confirm the “multiple hits theory” according to which there is a complex interaction between environmental and genetic factors contributing to the variable and incomplete penetrance of genetically based aHUS [8]. In support of this point, reports exist about adult patients with complement mutations developing aHUS after an infection; this could indicate that a trigger event, for example an infection with microbiological agents with complement-activating capabilities (including some influenza virus strains), is necessary for the manifestation of the disease in genetically susceptible individuals [17, 18, 19]. Our patient demonstrated signs of complement activation with low C3 and increased C5b-9 complex either on resting and ADP-activated endothelial cells [20], with subsequent restitutio ad integrum. After 18 months of follow-up, neither clinical nor biochemical signs of relapse were evident. Genetic screening for complementary factors mutations was anyhow performed, but turned out negative. These findings support the hypothesis of a direct role of the H1N1 virus in determining a transient complement activation in our patient. 

## Conclusions and future perspectives 

Understanding the pathogenetic mechanisms underlying influenza-associated HUS (iHUS) is crucial for acute and long-term management [8]. There are no recommendations for the treatment of iHUS, and the current therapeutic approach is based on supportive therapy, including dialysis and blood products, NA inhibitors (e.g., oseltamivir), and if needed plasma exchange (PLEX) and anticomplement therapy (e.g., eculizumab). The efficacy and safety of NA inhibitors for the management of influenza have been demonstrated in large clinical trials [21], but it could be interesting to investigate its possible efficacy to prevent iHUS based on the potential causative role of viral NA. PLEX and eculizumab, on the other hand, find their rationale in the evidence of a dysregulation of the complementary system associated to iHUS. So, at the time of diagnosis, an in-depth study of the complementary profile is recommendable to identify patients with APC dysregulation that could benefit from such therapies. 

## Funding 

There are no funders to report for this submission. 

## Conflict of interest 

The authors declare no conflict of interest. 
